# Evaluation of the left and right ventricular systolic and diastolic function in asthmatic children

**DOI:** 10.1186/s12872-016-0328-x

**Published:** 2016-07-08

**Authors:** Esra Akyüz Özkan, Hashem E. Khosroshahi

**Affiliations:** Department of Pediatrics, Bozok University Medical Faculty, Yozgat, Turkey; Department of Pediatric Cardiology, Bozok University Medical Faculty, Yozgat, Turkey

**Keywords:** Asthma, Children, Echocardiography, Tissue Doppler imaging, Ventricular function

## Abstract

**Background:**

Asthma is the most common cause of respiratory disorders among children. We aimed to investigate left (LV) and right (RV) ventricular function in asthmatic children as detected by conventional and tissue Doppler echocardiography.

**Methods:**

Fifty pediatric patients with asthma and forty healthy children were studied. Pulmonary function tests, electrocardiography and echocardiographic examinations were performed on all children.

**Results:**

Rate-corrected velocity of circumferential fiber shortening (VCFc) (*p* = 0.044), the ratio between heights of early and late diastolic flow velocity peaks (E/A) (*p* = 0.019) and LV end-systolic wall stress (ESWSm) was lower (*p* = 0.003), RV stroke volume (SV) (*p* = 0.002), LV SV (*p* = 0.001), tricuspid annular plane systolic excursion (TAPSE) (*p* = 0.034), tricuspid annular peak velocity during systole (S’) (*p* = 0.022), tricuspid and mitral early diastolic velocities (E’) (*p* = 0.012, *p* = 0.003 respectively) were lower in asthmatic children than controls. The mitral valve ejection time (ET) was high in asthmatic group (*p* = 0.027).

FEV1 was positively correlated with isovolumetric relaxation time (IVRT) (*p* = 0.018) (*r* = 0.382) and mitral ET (*p* = 0.018) (*r* = 0.381). PEF was negatively correlated with the RV work index (*p* = 0.032) (*r* = -0.348) and LV work index (*p* = 0.005) (*r* = -0.457).

**Conclusion:**

Although cardiac systolic function was found to be impaired in asthmatic patients, contrary to the literature, diastolic dysfunction was not observed in these patients, even by tissue Doppler imaging, and this finding may be attributed to using inhaled corticosteroid

## Background

Asthma is a chronic inflammatory disease of the airways which is related to airway obstruction, hyper-responsiveness and characterized recurrent wheezing, coughing and breathlessness [1]. Bronchial asthma affects many organs including the heart. There are many explanations for the occurrence of cardiac dysfunction in asthmatic children [2–4]. It is suggested that chronic hypoxia may cause pulmonary arterial hypertension, which causes RV hypertrophy and/or dilatation [2]. It is also suggested that recurrent hypoxemia and the release of various mediators and cytokines in bronchial asthma may cause chronic inflammation, which could induce pulmonary vasoconstriction [3, 4]. Other hypotheses concluded that the exaggerated respiratory efforts may raise intrathoracic pressure that increases RV afterload and consequently pulmonary hypertension with RV hypertrophy and/or dilatation. As a result, pulmonary hypertension occurs and leads to RV hypertrophy and/or dilatation [3–5]. As a result of chronic pressure overload, the RV hypertrophies and dilates and leads to both systolic and diastolic dysfunction [5]. The level of RV diastolic dysfunction depends on the degree of RV hypertrophy and total pulmonary resistance. Interaction between the RV and LV related to increased LV afterload and decreased LV preload, and thus LV dysfunction [6]. There are important limitations to determining RV function by conventional echocardiography [7]. The unavailability of the RV at the back the sternum leads to poor image quality. Also, locating the endocardial boundary of the anterior wall is a problem owing to the thicker trabeculations compared with the LV. Furthermore, the RV cavity has a more complex geometry than the LV, and RV performance depends on extrinsic conditions, such as afterload, preload, and LV performance.

Our study aimed to investigate ventricular diastolic and systolic functions, LV wall stresses and circumferential fiber shortening by using tissue Doppler imaging (TDI) and conventional echocardiography in asthmatic children, using regular inhaled corticosteroid, without any cardiovascular symptoms.

## Methods

We studied 50 pediatric patients (35 male and 15 female), selected randomly from those with bronchial asthma, and 40 healthy subjects. Inclusion criteria include all patients meeting the criteria for bronchial asthma [1]. Exclusion criteria were patients with comorbid diseases, such as upper or lower respiratory infection, allergic rhinitis, gastroesophageal reflux, obesity, chronic cardiovascular or pulmonary diseases and acute asthma attack during the last 4 weeks.

The control group was chosen from healthy individuals. The ethics committee of the institution approved the study and informed consent forms signed by the parents were obtained. All the children included in the study were subjected to full history taking. Complete physical examinations were performed by the same physician. Body height and weight, blood pressure (BP) and heart rate of all children were recorded. The patients and the controls who were >6 years underwent pulmonary function tests using spirometry forced expiratory volume in 1 s (FEV1), forced vital capacity (FVC), the ratio of FEV1 to FVC, and peak expiratory flow (PEF), which were all documented.

All asthmatic patients involved in this study were moderately asthmatic and received inhaled corticosteroid treatment for different periods of time and doses.

An electrocardiogram was simultaneously recorded for all patients. Transthoracic echocardiography was performed by a single experienced pediatric cardiologist, blinded to the subjects, and the following parameters were monitored: LV end-diastolic pressures (LVEDPs), LV mass (LVM, g) according to the formula of Devereux [8], stroke volume (SV), ejection fraction (EF, %), fractional shortening (FS, %), ratio between heights of early and late diastolic flow velocity peaks (E/A ratio) for both mitral and tricuspid valves, deceleration time (DT, ms), LV meridional end-systolic wall stress (ESWSm, g/cm^2^), Midwall Shortening Fraction (SFmid), heart rate corrected circumferential fiber shortening (VCFc), midwall VCFc, myocardial fiber stress (MFS, g/cm^2^), RV and LV work index (RVWI, LVWI respectively), LV and RV relative wall thickness (LVRWT, RVRWT, mm) mitral and tricuspid annular plane systolic excursion (MAPSE, TAPSE), RV Pre-ejection Period/RV ejection time (RVPEP/RVET), RV Pre-ejection period/RV acceleration time (RPEP/AT), and Acceleration time/Pulmonary artery ejection time (AT/PAET).

The following parameters were monitored by TDI: annular peak velocity during late diastole (A’) annular peak velocity during early diastole (E’), isovolumetric relaxation time (IVRT), isovolumetric contraction time (IVCT), myocardial performance index (MPI), annular peak velocity during systole (S’), ejection time (ET), time velocity integral for aortic valve (Aort VTI), time velocity integral for pulmonary artery (PA VTI), mitral valve myocardial acceleration during isovolumic contraction (IVMA-MV), tricuspid valve myocardial acceleration during isovolumic contraction (IVMA-TV), and pulmonary artery myocardial acceleration during isovolumic contraction (IVMA-PA).

EF, SV, LVOT and AoVTI were calculated as described in previous studies [9].

LV fractional shortening (FS) was calculated as LVVD-LVDS/LVDD [10].

Mitral and tricuspid filling velocities were recorded from the apical four-chamber view with the pulse-wave Doppler during diastole. E/A and DT were used as both ventricular diastolic function parameters. The ratios of E to A were calculated for mitral and tricuspid valves [11].

The Doppler-derived myocardial performance index (MPI) for LV and RV, combining systolic and diastolic time intervals, were calculated IVCT+ IVRT/ET [12].

VCFc (circ/s) = (SF x (1500/heart rate)^0.5^/LV ET)

Midwall VCFc was calculated as = 0.0007xMFS + 0.65

ESWSm was calculated by the method of Grossman et al [12] and MFS according to the formula recommended by Regen [13].

SFmid = [(LVED + h_d_/2 + s_d_/2) – LVES – *mwst*]/(LVED+ h_d_/2 + s_d_/2)

Where the *mwst* calculared as = [(LVED + (h_d_ + s_d_)/2)^3^ - LVED^3^ + LVES^3^)^0,333^ –LVES]

h_d_ : left ventricular end-diastolic posterior wall thickness

s_d_: end-diastolic septal thickness

LVES stands for LV end systolic dimension and LVED stands for LV end diastolic dimension.

LVEDP = 21.6 (Q-M1/A2-OS) + 1.1, OS: opening snap of the mitral valve, A2: aortic component of the second heart sound, Q: Q wave on the ECG, M1: mitral component of the first heart sound [14].

RV and LV function were also evaluated using TDI: Peak systolic (S’) and early and late diastolic velocities (E’ and A’) were measured from the apical four-chamber view with the pulsed-wave Doppler sample volume placed at the tricuspid and mitral annulus.

Cardiac time intervals comprising RV included IVCT from the end of the tricuspid flow to the beginning of the pulmonary flow, IVRT from the end of the pulmonary flow to the beginning of the tricuspid flow, and ET from the beginning to the end of the pulmonary flow were also measured.

### Statistical analysis

The statistical analyses were performed by the Statistical Package for Social Sciences (SPSS). Kolmogorov–Smirnov and Shapiro Wilk tests were used to assess normality of distribution.

Parametric variables were compared using the Student’s *t* test for normally distributed data and the Mann Whitney *u* test for not normally distributed data. Bivariate associations of the variables were assessed using Pearson’s correlation coefficients. Variables were expressed as mean ± SD and *p* value <0.05 was considered to indicate statistical significance.

## Results

The current study enrolled 50 patients (35 boys and 15 girls) with a mean age of 10.56 ± 3.03 years who had diagnosis of bronchial asthma. The characteristics of the children and healthy subjects are shown in Table 1. The spirometry findings are summarized in Table 2.

**Table 1:** Comparison (mean ± SD) of clinical characteristics

Variable	asthmatic children	Healthy control	*p* value
	*n* = 50	*n* = 40	
Age (years)	10.56 ± 3.03	12.08 ± 3.21	0.250^a^
Male/female	35/15	17/23	0.314^c^
Body height (cm)	142.8 ± 12.2	144.5 ± 12.4	0.120^b^
Body weight (kg)	28.6 ± 12.5	28.6 ± 12.5	0.480^b^
Heart rate (bpm)	84.68 ± 14.93	86.42 ± 10.87	0.537^a^
Systolic BP (mmHg)	104.74 ± 8.94	103.78 ± 9.47	0.345^b^
Diastolic BP (mmHg)	64.02 ± 5.88	61.38 ± 9.63	0.175^b^

^a^ Student’s t test, ^b^ Man-Whitney u test, ^c^ Chi-square test

**Table 2:** Comparison (mean ± SD) of spirometry findings

Variable	asthmatic children	Healthy controls	*p* value^*a*^
	*n* = 50	*n* = 40	
PEF	90.02 ± 9.7	92.7 ± 8.9	0.320
FEV1	82.8 ± 12.2	92.2 ± 9.3	**0.002** ^*^
FVC	92.1 ± 8.5	95.2 ± 11.4	0.123
FEV1/FVC	89.6 ± 9.7	96.2 ± 10.5	0.012*

^*a*^ Student’s *t* test

*Correlation is significant at the 0.05 level

**Fig. 1:**
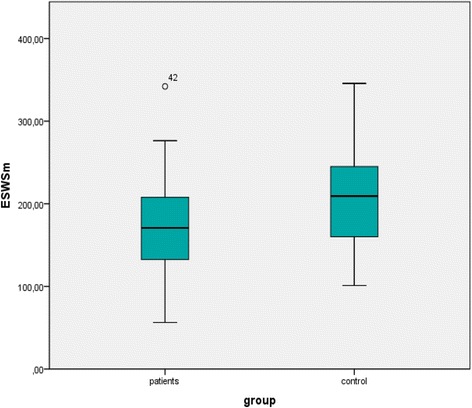
Distribution of LV meridional end-systolic wall stress (ESWSm) between groups

LV and RV echocardiographic findings are listed in Tables 3 and 4. The rate-corrected velocity of circumferential fiber shortening (VCFc) (*p* = 0.044), E/A ratio (*p* = 0.019), ESWSm was lower (*p* = 0.003), RSV (*p* = 0.002), LSV (*p* = 0.001), tricuspid annular peak velocity during systole (S’) (*p* = 0.022), TAPSE (*p* = 0.034) and tricuspid and mitral E’ (*p* = 0.012, *p* = 0.003 respectively) were lower in asthmatic children than control subjects. Mitral valve ET was higher in the asthmatic group (*p* = 0.027). Distribution of ESWSm between groups is showed in Fig. 1. TDI parameters are listed in Table 5.

**Table 3:** Comparison (mean ± SD) of left ventricular echocardiographic findings

Variable	Asthmatic children	Healthy controls	*p* value
	50 (M/F = 35/15)	40 (M/F = 17/23)	
LSV (ml)	40.75 ± 14.26	50.99 ± 13.08	0.001^a^
LVWT (cm)	0,40 ± 0,087	0,38 ± 0,055	0,172^a^
LV-EF (%)	0.66 ± 0.1	0.63 ± 0.07	0.182^a^
DT (msn)	184.79 ± 53.98	192.50 ± 45.60	0.465^a^
LV-FS (%)	0.36 ± 0.08	0.34 ± 0.05	0.185^a^
LVM (gr)	67.03 ± 22.89	69 ± 18.26	0.440^b^
E/A	1.78 ± 0.27	1.93 ± 0.32	0.019^a^
L-sist (ms)	340.34 ± 42.89	346.83 ± 28.01	0.485^b^
MAPSE (cm)	1.09 ± 0.18	1.07 ± 0.17	0.672^b^
LVEDP (mmHg)	26.45 ± 5.11	26.96 ± 4.63	0.451^b^
LVWI (gm-m/m^2^)	35.61 ± 9.41	33.32 ± 7.24	0.387^b^
WSM (g/cm^2^)	1114.18 ± 595.42	1356.16 ± 562.54	0.053^a^
ESWSm (g/cm^2^)	172.5 ± 58.05	209.22 ± 55.53	0.003^a^
VCFc (circ/s)	0.0051005 ± 0.00	0,0057375 ± 0.00	0.044^b^
Sfmid (%)	-0.36 ± 0.10	-0.38 ± 0.07	0.257^b^
Fiber stress (g/cm^2^)	36.55 ± 9.32	39.74 ± 11.61	0.160^b^
Midwall VCFc (circ/s)	0.67 ± 0.01	0.68 ± 0.01	0.160^b^

*LSV* Left ventricle stroke volume, *LVWT* Left ventricular wall thickness, *EF* Ejection fraction, *DT* Deceleration time, *LV_FS* Left ventricle fractional shortening, *LVM* Left ventricular mass, *A* Peak velocity during late diastole, *E* Peak velocity during early diastole, *L-sist T* Left Systolic Time (Contraction time), *MAPSE* Mitral annular plane systolic excursion; *LVEDP* Left Ventricle end-diastolic Pressure, *LVWI* Left ventricular work index; *WSM* Meridional LV wall stress, *ESWSm* Meridional end-systolic wall stress, *VCFc* Rate-corrected velocity of circumferential fiber shortening

^a^ Student’s *t* test, ^b^ Mann-Whitney *u* test

**Table 4:** Comparison (mean ± SD) of right ventricular echocardiographic findings

Variable	asthmatic children	Healthy controls	*p* value
	50 (M/F = 35/15)	40 (M/F = 17/23)	
RSV (ml)	50.01 ± 18.26	64.70 ± 23.66	0.002^b^
RVWT (cm)	0,47 ± 0,11	0,47 ± 0,10	0.980^a^
RV-EF (%)	0.73 ± 0.15	0.71 ± 0.13	0.385^b^
DT (msn)	198.19 ± 62.73	189.87 ± 46.76	0.490^b^
RV-FS (%)	0.42 ± 0.12	0.39 ± 0.10	0.372^a^
E/A	1.72 ± 0.26	1.80 ± 0.33	0.243^a^
R-sist (ms)	362.78 ± 32.74	362.72 ± 28.90	0.807^b^
TAPSE (cm)	2.02 ± 0.20	2.15 ± 0.32	0.034^b^
RV WI (gm-m/m^2^)	4.24 ± 1.20	4.57 ± 1.68	0.572^b^
RVPEP/RVET (ms)	0.19 ± 0.04	0.19 ± 0.03	0.767^b^
RPEP/AT (ms)	0.50 ± 0.13	0.51 ± 0.11	0.679^b^
AT/PAET (ms)	0.39 ± 0.06	0.38 ± 0.05	0.372^b^

*RSV* Right ventricle stroke volume, *RVWT* Right ventricular wall thickness, *RV-EF* Right ventricle ejection fraction; *RV-FS* Right ventricle fractional shortening, *A* Peak velocity during late diastole, *E* Peak velocity during early diastole, *DT* Deceleration Time, *R-sist T* Right ventricle systolic time (contraction time), *TAPSE* Tricuspid annular plane systolic excursion, *RVWI* Right ventricular work index, *RVPEP/RVET* Right Ventricle Pre-Ejection Period/Right ventricle Ejection Time, *RPEP/AT* Right ventricle Pre-Ejection Period/Right ventricle Acceleration Time, *AT/PAET* Acceleration time/Pulmonary artery Ejection Time

^a^ student’s *t* test, ^b^ Mann-Whitney *u* test

**Table 5:** TDI findings (mean ± SD) of the patients and control group

Variable	asthmatic children	Healthy controls	*p* value
	50 (M/F = 35/15)	40 (M/F = 17/23)	
Mitral annulus			
E’(m/sn)	0.17 ± 0.03	0.19 ± 0.03	0.003^a^
A’(m/sn)	0.08 ± 0.02	0.12 ± 0.21	0.635^b^
S’(m/sn)	0.072 ± 0.022	0.077 ± 0.013	0.147^a^
Aort-VTI (cm)	21.96 ± 3.91	22.48 ± 3.60	0.514^a^
IVCT (ms)	65.89 ± 11.57	69.05 ± 15.08	0.533^a^
IVRT (ms)	64.13 ± 8.87	64.05 ± 8.48	0.880^b^
ET-MV (ms)	265.53 ± 40.58	245.80 ± 30.90	0.027^b^
IVMA-MV (m/s^2^)	0.07 ± 0.03	0.08 ± 0.04	0.575^b^
Mitral-MPI (ms)	0.50 ± 0.09	0.54 ± 0.10	0.089^b^
Tricuspid annulus			
E’ (m/sn)	0.17 ± 0.02	0.18 ± 0.02	0.012^b^
A’(m/sn)	0.11 ± 0.04	0.12 ± 0.03	0.968^b^
S’(m/sn)	0,08 ± 0,022	0,10 ± 0,028	0.022^a^
PA-VTI (cm)	21.87 ± 2.73	22.39 ± 2.80	0.377^a^
IVCT (ms)	66.63 ± 10.87	66.40 ± 13.07	0.517^b^
IVRT (ms)	65.74 ± 9.77	64.35 ± 8.26	0.436^b^
ET-TV (ms)	253.79 ± 36.58	241.10 ± 30.01	0.118^b^
IVMA-TV(m/s^2^)	0.09 ± 0.04	0.08 ± 0.04	0.955^b^
IVMA-PA(m/s^2^)	0.10 ± 0.17	0.08 ± 0.031	0.557^a^
Tricuspit MPI (ms)	0.53 ± 0.08	0.56 ± 0.09	0.070^a^

*A*’ Annular peak velocity during late diastole, *E*’ Annular peak velocity during early diastole; *IVRT* Izovolumetric relaxation time, *IVCT* Izovolumetric contraction time, *MPI* Myocardial performance index, *S*’ Annular peak velocity during systole, *ET* Ejection time, *Aort VTI* Time velocity integral for aortic valve, *PA VTI* Time velocity integral for pulmonary artery, *IVMA-MV* Mitral valve myocardial acceleration during izovolumetric contraction, *IVMA-TV* Tricuspid valve myocardial acceleration during izovolumetric contraction, *IVMA-PA* Pulmonery artery myocardial acceleration during izovolumetric contraction

^a^ student’s *t* test, ^b^ Mann-Whitney *u* test

We performed pulmonary function tests on patient and control groups and correlated FEV1, FVC, FEV1/FVC and PEF with cardiac parameters. There were no differences between the asthmatic and control group. In correlation analysis, FEV1 was positively correlated with IVRT (*p* = 0.018) (*r* = 0.382) and ET-MV (*p* = 0.018) (*r* = 0.381). PEF was negatively correlated with RVWI (*p* = 0.032), (*r* = -0.348) and LVWI (*p* = 0.005), (*r* = -0.457).

## Discussion

Asthma is the most common cause of respiratory disability in children [3]. We aimed to investigate both ventricular function in children with bronchial asthma as detected by conventional echocardiography and TDI.

Our results confirms the results reported by Shedeed et al [15] and Mahmoud et al [16] who found no statistically significant differences between asthmatic children and controls regarding heart rate and systolic and diastolic BP.

According to some studies right ventricular (RV) systolic and diastolic dysfunction were detected, even in mild cases, by echocardiography. On the other hand, left ventricular (LV) dysfunction usually presents itself in severe asthmatic cases which may be reversible in acute conditions [17, 18].

Some studies showed that there were no differences in echocardiographic findings regarding the ventricular function with the exception of RV wall thickness [15, 19]. We couldn’t find any differences between the two groups regarding RVWT and LVWT.

IVMA, MPI, EF, FS, TAPSE, MAPSE, SV, VCFc and ESWSm values of both ventricles were evaluated to assess ventricular systolic function. IVMA, MPI, EF, FS and MAPSE were similar in both groups. TAPSE was lower in asthmatic patients.

IVMA is a measurement of ventricular contractile function that is unaffected by preload and afterload changes in a physiological range [20]*.* In this study we couldn’t show any differences between IVMA values in either group.

The SV of both the RV and LV were lower in asthmatic children than in the controls in our study. Hedlin et al [21] reported that SV decreased in asthmatic patients whose asthma was provoked by exercise and increased with inhale salbutamol.

To assess LV contractility, we also analyzed changes in LV VCFc and afterload (ESWSm) [12]. The ventricular contractility and myocardial performance may have been affected by chamber geometry, which need to be identified by measuring ESWSm, VCFc-midwall and MFS. ESWSm was accepted as afterload (no more shortening point). ESWSm, which is dependent on both chamber shape and mass/volume ratio, demonstrates the forces opposing predominantly meridional and circumferential planes. This is an index of total forces per unit of myocardium, and therefore may cause an underestimation in true afterload. MFS, as representative of myofiber afterload, is a more accurate index of afterload in hypertrophied or dilated LV [22]. In the current study, ESWSm and VCFc were low, while MFS was similar in the asthmatic group. SFmid as a systolic ejection index of deeper layers of myocardium provides more physiologically appropriate measurements of LV in wall thickness and conditions like LV concentric hypertrophy and provides information to assess the myocardial performance [22]. In this study, SFmid was similar in both groups. As an indication of compromised LV contractility, LV ESWSm was significantly lower in asthmatic patients.

To evaluate ventricular diastolic function we used mitral and tricuspid E/A, E’, A’, DT, IVRT and IVCT by using conventional echocardiography and TDI. There are some studies which reported RV and LV diastolic dysfunction in patients with moderate to severe asthma [2, 23, 24]. In contrast with these studies, we turned away from the diagnosis of diastolic dysfunction in asthmatic patients because both mitral and tricuspid E/A values were >1 without any sign of pseudo-normal E/A values.

Mitral and tricuspid annulus early diastolic velocities were different by TDI. Mitral E’, tricuspid E’ and S’ were lower in the asthmatic group. There were no significant differences among the IVCT, IVRT and DT in either the mitral or tricuspid annulus. These findings showed that there was no diastolic dysfunction among our asthmatic children even by TDI. This result may due to use of inhaled corticosteroids. Shedeed [15] reported that E’, A’, and S’ were significantly lower, and E’/A’, IVRT, and IVCT significantly greater in asthmatic children as detected by TDI. Zeybek et al [18] showed that lateral annular velocities of tricuspid and IVRT were different between asthmatic children and controls. Abdalla et al [25] suggested that LV diastolic function is impaired in patients with bronchial asthma despite there being no effect on RV diastolic function. Ozdemir et al [19] revealed the same, although there was no difference by conventional echocardiography in asthmatic children. TDI showed subclinical dysfunction of the right ventricle and they suggested that TDI was useful in the early detection of some detrimental effects of asthmatic cases.

Hirschfeld et al [26] measured the RPEP and RVET in 64 pediatric patients undergoing cardiac catheterization and demonstrated a strong correlation between pulmonary artery diastolic pressure and the ratio RPEP/RVET (*r* = 0.72). In one study it has been suggested that normal RPEP/RVET was not significantly influenced by age or heart rate, and therefore could be used as an index of RV performance throughout a wide range of pediatric age groups. On the other hand, the same study revealed that the RPEP/RVET ratio correlated with mean pulmonary artery pressure and was associated with pulmonary vascular resistance [27]. The current study failed to demonstrate any differences regarding the RVPEP/RVET ratio between the groups.

We performed pulmonary function tests in both groups. FEV1 was positively correlated with IVRT and ET-MV. PEF was negatively correlated with RVWI and LVWI. Ozdemir et al [19] reported that PEF was negatively correlated with tricuspid E’/A’, while Sheeded et al [15] demonstrated a negative correlation between PEF and IVRT.

Inhaled corticosteroids exert a strong anti-inflammatory effect on airways; they represent the most effective agents for long-term asthma control [28]. Some studies showed that inhaled corticosteroids have protective effects on cardiac function in asthmatic patients [29]. In the current study, all the children were using inhaled corticosteroids with a different period of time and doses and this study showed that, even with this protective effect, myocardial function can be impaired in asthmatic children.

In our previous study on asthmatic children who use inhaled corticosteroid, arterial stiffness were decreased, distensibility and strain were increased [30]. We suggested that using inhaled corticosteroids could have provided certain protective effects in asthmatic children. A proposed mechanism that may explain the decrease in arterial stiffness involves nitric oxide synthesis and vasodilatation [31].

## Conclusion

Asthma affects ventricular contractility in children. Although cardiac systolic function was found to be impaired in asthmatic patients, contrary to the literature diastolic dysfunction didn’t observed in these patients even by tissue Doppler imaging, may be due to using inhaled corticosteroid. This was one of the most comprehensive studies among asthmatic patients. Further studies with larger sample sizes or without using corticosteroid treatment warranted to better elucidate the cardiac function in asthmatic children.

## Abbreviations

A, Peak velocity during late diastole; A’, Annular peak velocity during late diastole; Aort VTI, Time velocity integral for aortic valve; AT/PAET, Acceleration time/Pulmonary artery Ejection Time; BP, Blood pressure; DT, Deceleration time; E, Peak velocity during early diastole; E’, Annular peak velocity during early diastole; EF, Ejection fraction; ESWSm, Meridional end-systolic wall stress; ET, Ejection time; FEV1, Forced expiratory volume in 1 s; FVC, Forced vital capacity; IVCT, Izovolumetric contraction time; IVMA-MV, Mitral valve myocardial acceleration during izovolumetric contraction; IVMA-PA, Pulmonery artery myocardial acceleration during izovolumetric contraction; IVMA-TV, Tricuspid valve myocardial acceleration during izovolumetric contraction; IVRT, Izovolumetric relaxation time; L-sist T, Left Systolic Time (Contraction time); LSV, Left ventricle stroke Volume; LVEDP, Left Ventricle end-diastolic Pressure; LV-FS, Left ventricle fractional shortening; LVM, Left ventricular mass; LVWI, Left ventricular work index; LVWT, Left ventricular wall thickness; MAPSE, Mitral annular plane systolic excursion; MFS, Myocardial fiber stress; MPI, Myocardial performance index; PA VTI, Time velocity integral for pulmonary artery; PEF, Peak expiratory flow; RPEP/AT, Right ventricle Pre-Ejection Period/Right ventricle Acceleration Time; R-sist T, Right ventricle systolic time (contraction time); RSV, Right ventricle stroke volume; RV-EF, Right ventricle ejection fraction; RV-FS, Right ventricle fractional shortening; RVPEP/RVET, Right ventricle Pre-Ejection Period/Right ventricle Ejection Time; RVWI, Right ventricular work index; RVWT, Right ventricular wall thickness; S’, Annular peak velocity during systole; TAPSE, Tricuspid annular plane systolic excursion; TDI, Tissue Doppler imaging; VCFc, Rate-corrected velocity of circumferential fiber shortening; WSM, Meridional LV wall stress.
